# Safety and effectiveness of intravenous tranexamic acid for posterior lumbar interbody fusion in patients on preoperative anticoagulants: a retrospective case–control study

**DOI:** 10.3389/fmed.2026.1729718

**Published:** 2026-04-24

**Authors:** Bing Qian, Hongqi Zhan, Shenshen Hao, Xiaoya Zhu, Fangshu Zhang, Chunzhi Gao, Yangyang Liu, Shengli Dong, Shuai Liu, Hongke Li, Fei Chen

**Affiliations:** 1 Emergency Medicine Department, Honghui Hospital, Xi’an Jiaotong University, Xi'an, Shaanxi, China; 2 Affiliated Hospital of Qingdao University, Qingdao, Shandong, China; 3 Department of Spine, Affiliated Hospital of Qingdao University, Qingdao, Shandong, China; 4 Department of Spine and Bone Oncology, General Hospital of Pingmei Shenma Medical Group, Pingdingshan, Henan, China; 5 Interventional Operating Room, Pingdingshan the Second People's Hospital, Pingdingshan, Henan, China; 6 Department of Orthopedics, Henan Provincial Third People's Hospital, Zhengzhou, Henan, China

**Keywords:** anticoagulants, hemostatic effect, posterior lumbar interbody fusion, safety, tranexamic acid

## Abstract

**Background:**

Tranexamic acid (TXA) is a hemostatic agent used in posterior lumbar interbody fusion (PLIF). However, reports on its use in patients on preoperative anticoagulants are scarce. This study evaluates the safety and efficacy of preoperative 1 g TXA in such patients.

**Methods:**

This study retrospectively analyzed the medical records of patients who underwent PLIF and had received anticoagulants within 1 week before surgery between January 2021 and June 2023. After general anesthesia, at 15 min before skin incision, patients who received intravenous TXA were recorded as the observation group (*n* = 31), and those who did not were recorded as the control group (*n* = 29). The main observation indicators included surgical time, intraoperative blood loss (IBL), postoperative drainage volume (PDV), number of blood transfusions, red blood cell count (RBC), hemoglobin (HB), and hematocrit (HCT) on the postoperative 1st, 4th, 7th, and last-test days. The secondary observation indicators included postoperative incision healing, number of lower limb deep vein thrombosis (DVT), prothrombin time (PT), activated partial thromboplastin time (APTT), thrombin time (TT), fibrinogen (FIB), platelet count (PLT) and postoperative hospital stay.

**Results:**

The surgeries were successfully completed. The incisions healed as grade A, and no DVT occurred. The IBL and PDV in the observation group were significantly lower than those in the control group (*p* < 0.05). There were no significant differences between the two groups in surgical time or blood transfusions (*p* > 0.05). The multivariate linear regression analysis revealed that the surgical segment and TXA were independent influencing factors for IBL and PDV, while age, body mass index, gender, coexisting hypertension, disease type, and anticoagulant type were not. Subgroup multivariate analysis further confirmed that anticoagulant type was not an independent influencing factor of IBL or PDV. There were no statistically significant differences between the two groups in RBC, HB, or HCT at any postoperative time point (*p* > 0.05). There was no significant difference in postoperative PT, APTT, TT, FIB, PLT, or postoperative hospital stay between the two groups (*p* > 0.05).

**Conclusion:**

For patients undergoing PLIF with preoperative anticoagulation, TXA administration appears safe and significantly reduces perioperative blood loss.

## Background

Posterior lumbar interbody fusion (PLIF) is one of the common surgeries for treating lumbar disc herniation (LDH), lumbar spinal stenosis (LSS), and lumbar spondylolisthesis (LS) (1). However, this surgery has the drawback of excessive intraoperative blood loss (IBL) and postoperative drainage volume (PDV) (2). Tranexamic acid (TXA), a synthetic lysine derivative, is a safe and effective antifibrinolytic hemostatic drug used in PLIF (3, 4). It can competitively bind to the lysine binding site of plasminogen, inhibit plasminogen activation, and thus exert antifibrinolytic and hemostatic effects (5, 6). The application methods of TXA include topical medication, intravenous medication, and combination therapy of the two ways. A single intravenous dose of 1 g TXA at 15 min before surgery is one of the recommended methods in the 2019 Chinese Expert Consensus (7). There are two advantages to this method. Firstly, preoperative intravenous medication is simple and feasible, and it does not interfere with anesthesia (8). Secondly, 15 min after administration, the drug can reach the surgical incision site and exert its hemostatic effect (9).

However, in clinical practice, there exists a non-negligible phenomenon that some patients undergoing PLIF are required to continue anticoagulant therapy preoperatively. This clinical reality presents a potential conflict with the surgical procedure itself. Aspirin is a commonly used anticoagulant. Due to its potential to increase IBL (10), an alternative anticoagulant therapy is often needed before surgery. The commonly used alternative is low molecular weight heparin (LMWH) (11). In addition, indobufen is an oral anticoagulant with an effect equivalent to that of an equal dose of aspirin (12), making it a suitable alternative for patients who are unwilling to receive subcutaneous injections of LMWH (13). Meanwhile, our previous studies found that preoperative administration of anticoagulants does not compromise the hemostatic efficacy of TXA in patients undergoing PLIF (13). Moreover, no significant differences were observed between LMWH and indobufen regarding their effects on IBL and PDV of PLIF (14). However, there is a paucity of research investigating the application of TXA in patients undergoing PLIF who are on pre-existing anticoagulant therapy. Thus, the safety and efficacy of TXA in this specific patient population remain to be elucidated, which constitutes a topic warranting further investigation.

Therefore, we conducted the present study with two primary objectives. The first was to evaluate the safety of TXA in patients receiving anticoagulant therapy before PLIF, and the second was to assess its hemostatic efficacy.

## Method

### Study design

This study was a single-center, retrospective case–control study. The study enrolled patients between January 2021 and June 2023. The inclusion criteria included the following points. *a*. Preoperative diagnosis was lumbar degenerative disease, including LDH, LSS, and LS. *b*. The patient received anticoagulant drugs, including LMWH or indobufen tablets, within 1 week before surgery. *c*. The surgical method was PLIF. *d*. The surgical segment was one to three segments. *e*. The anesthesia method was general anesthesia. The exclusion criteria included the following points. *a*. The patient had a hematologic disease before surgery. *b*. The patient had diabetes before the operation. *c*. The patient had a history of lumbar spine fracture, tuberculosis, tumor or surgery. *d*. There was a history of deep vein thrombosis (DVT) in the lower limbs before surgery. *e*. Perioperative cerebrospinal fluid leakage occurred.

Data collection and analysis were performed by independent, trained personnel. A total of 60 patients met the inclusion criteria and were enrolled in this study. Of these, 23 were male and 37 were female, with a mean age of 64.03 ± 9.24 years (range: 39–80 years). The grouping criterion was preoperative intravenous administration of TXA. 31 patients who received an intravenous dose of 1 g TXA 15 min after induction of general anesthesia (prior to skin incision) were classified as the observation group. 29 patients who did not receive TXA were included in the control group.

Baseline data included age, gender, body mass index (BMI), coexisting hypertension, surgical segment, disease type, prothrombin time (PT), activated partial thromboplastin time (APTT), thrombin time (TT), fibrinogen (FIB), platelet count (PLT), hemoglobin (HB), red blood cell count (RBC), hematocrit (HCT). There were no significant differences between the two groups in baseline data. They are presented in Table 1.

**Table 1:** Comparison of baseline data between the two groups.

Groups	Observation group (*n* = 31)	Control group (*n* = 29)	*t*/*χ*^2^/*Z*	*p*
Age, year	63.968 ± 9.083	64.103 ± 9.571	−0.056	0.955
Gender, *n*			1.001	0.317
Male	10	13		
Female	21	16		
BMI, kg/m^2^	24.618 ± 3.101	25.463 ± 3.057	−1.063	0.292
Disease type, *n*			0.222	0.895
LDH	4	5		
LSS	19	17		
LS	8	7		
Surgical segment, *n*			1.157	0.561
One	12	14		
Two	16	11		
Three	3	4		
Coexisting hypertension, *n*			0.534	0.465
Yes	9	11		
No	22	18		
PT, s	11.194 ± 0.673	10.869 ± 0.750	1.766	0.083
APTT, s	31.106 ± 2.741	30.845 ± 3.290	0.385	0.738
TT, s	14.50 [13.90; 15.10]	15.00 [14.22; 16.06]	−1.894	0.058
FIB, g/L	2.99 [2.63; 3.32]	2.93 [2.65; 3.15]	−0.436	0.663
PLT, 10^9^/L	234.742 ± 53.542	227.931 ± 65.089	0.444	0.659
RBC, 10^12^/L	4.205 ± 0.424	4.372 ± 0.420	−1.533	0.131
HB, g/L	130.903 ± 14.152	136.000 ± 14.350	−1.385	0.171
HCT, L/L	0.39 [0.37; 0.42]	0.41 [0.38; 0.43]	−1.640	0.101

### Research method

Patients in both groups were placed in the prone position. After general anesthesia, the observation group received an intravenous dose of 1 g TXA 15 min before skin incision, while the control group did not receive TXA. All surgeries were performed using standard PLIF. Two negative pressure drainage tubes were placed before suturing the incision. The criteria for drain removal was a drainage volume of less than 50 mL / 24 h. Postoperatively, the same routine therapeutic measures were implemented. Concurrently, the incision healing status and the presence of lower limb DVT were monitored.

### Outcome indicators

The main observation indicators included surgical time, IBL, PDV, number of blood transfusions, RBC, HB, and HCT on the 1st, 4th, 7th, and last-test days after surgery. The secondary observation indicators included postoperative incision healing, number of lower limb DVT, PT, APTT, TT, FIB, PLT, and postoperative hospital stay.

### Statistical methods

The data analysis was conducted with R software (version 4.4.2). Continuous variables with a normal distribution were expressed as mean ± standard deviation, and an independent samples *t*-test was used for comparison between the two groups. Non-normally distributed continuous variables were expressed as median [interquartile range, IQR] (P25, P75), and the Mann–Whitney *U* test was used for comparison between the two groups. The Chi-square test was used to compare categorical variables between the two groups. Multiple linear regression analysis was performed using the lm function in R. The result variable with significant differences was taken as the dependent variable. Age, BMI, gender, coexisting hypertension, disease type, anticoagulant type, surgical segment, and TXA were used as independent variables. The enter method was adopted for the screening of independent variables. When the independent variable was a categorical variable, the group with the minimum value was used as the reference group. When the independent variable was a continuous variable, it was directly included in the linear regression model. A mixed-effects model for repeated measures was used to compare RBC, HB, and HCT at different postoperative time points. *p* < 0.05 was considered statistically significant.

## Results

The surgeries of both groups were successfully completed. The postoperative incisions healed as grade A in both groups, and no DVT occurred. The IBL and PDV in the observation group were lower than those in the control group, and the differences were statistically significant (*p* < 0.05). There were no significant differences between the two groups in surgical time or number of blood transfusions (*p* > 0.05). The results are shown in Table 2.

**Table 2:** Comparison of main observation indicators between the two groups.

Groups	Observation group (*n* = 31)	Control group (*n* = 29)	*t*/*χ*^2^/*Z*	*p*
Surgical time, min	192.419 ± 45.804	195.414 ± 52.370	−0.236	0.814
IBL, mL	300 [300; 500]	500 [300; 800]	−2.177	0.030
PDV, mL	220 [200; 310]	330 [310; 400]	−4.037	0.000
Number of blood transfusions, *n*			0.548	0.459
Yes	7	9		
No	24	20		

The results of the multiple linear regression analysis, with IBL and PDV as dependent variables, were as follows. The surgical segment and TXA were independent influencing factors for IBL and PDV, while age, BMI, gender, coexisting hypertension, disease type, and anticoagulant type were not. These findings are presented in Tables 3 and 4.

**Table 3:** Multivariate linear regression analysis with IBL as the dependent variable.

Variables	*B*	SE	*t*	*p*	95% CI	
					Lower bound	Upper bound
Constant	1008.619	437.302	2.306	0.025	129.363	1887.874
Age, year	1.626	3.429	0.474	0.637	−5.268	8.521
BMI, kg/m^2^	−6.959	10.277	−0.677	0.502	−27.623	13.705
Gender (1 = male, 0 = female)	53.318	64.559	0.826	0.413	−76.487	183.123
Coexisting hypertension (1 = yes, 0 = no)	112.191	69.890	1.605	0.115	−28.333	252.714
TXA (1 = yes, 0 = no)	−185.744	57.307	−3.241	0.002	−300.967	−70.522
Anticoagulant type (1 = LMWH, 0 = indobufen)	−83.708	64.975	−1.288	0.204	−214.349	46.934
Disease type (reference: LS)						
LSS	−47.752	71.596	−0.667	0.508	−191.705	96.201
LDH	−8.467	94.336	−0.090	0.929	−198.143	181.208
Surgical segment (reference: three)						
One	−586.691	98.686	−5.945	0.000	−785.113	−388.269
Two	−454.751	96.963	−4.690	0.000	−649.709	−259.794

**Table 4:** Multivariate linear regression analysis with PDV in the observation group as the dependent variable.

Variables	*B*	SE	*t*	*p*	95% CI	
					Lower bound	Upper bound
Constant	272.321	170.631	1.596	0.117	−70.756	615.398
Age, year	1.137	1.338	0.850	0.400	−1.553	3.827
BMI, kg/m^2^	−1.500	4.010	−0.374	0.710	−9.563	6.563
Gender (1 = male, 0 = female)	−8.339	25.190	−0.331	0.742	−58.988	42.310
Coexisting hypertension (1 = yes, 0 = no)	−0.885	27.270	−0.032	0.974	−55.716	53.946
TXA (1 = yes, 0 = no)	−105.785	22.360	−4.731	0.000	−150.743	−60.826
Anticoagulant drug type (1 = LMWH, 0 = indobufen)	11.340	25.353	0.447	0.657	−39.635	62.316
Disease type (reference: LS)						
LSS	48.322	27.936	1.730	0.090	−7.847	104.491
LDH	65.158	36.809	1.770	0.083	−8.851	139.168
Surgical segment (reference: three)						
One	−27.874	38.506	−0.724	0.473	−105.296	49.548
Two	53.179	37.834	1.406	0.166	−22.892	129.249

In the observation group, multiple linear regression analysis with IBL as the dependent variable revealed that the surgical segment was an independent influencing factor of IBL, whereas age, BMI, gender, coexisting hypertension, anticoagulant type, and disease type were not. In the analysis with PDV as the dependent variable, none of the aforementioned independent variables emerged as independent influencing factor. The results are presented in Tables 5 and 6.

**Table 5:** Multivariate linear regression analysis with IBL as the dependent variable in the observation group.

Variables	*B*	SE	*t*	*p*	95% CI	
					Lower bound	Upper bound
Constant	914.864	505.332	1.810	0.085	−136.031	1965.758
Age, year	0.605	4.087	0.148	0.884	−7.894	9.104
BMI, kg/m^2^	−8.401	11.752	−0.715	0.483	−32.842	16.039
Gender (1 = male, 0 = female)	65.872	68.405	0.963	0.347	−76.385	208.129
Coexisting hypertension (1 = yes, 0 = no)	127.176	87.181	1.459	0.159	−54.126	308.478
Anticoagulant type (1 = LMWH, 0 = indobufen)	−74.696	81.244	−0.919	0.368	−243.653	94.260
Disease type (reference: LS)						
LSS	−70.271	78.100	−0.900	0.378	−232.688	92.146
LDH	135.256	114.357	1.183	0.250	−102.563	373.076
Surgical segment (reference: three)						
One	−558.974	119.437	−4.680	0.000	−807.357	−310.592
Two	−584.464	112.982	−5.173	0.000	−819.422	−349.506

**Table 6:** Multivariate linear regression analysis with PDV as the dependent variable in the observation group.

Variables	B	SE	t	p	95% CI	
					Lower bound	Upper bound
Constant	221.184	259.678	0.852	0.404	−318.846	761.213
Age, year	−0.054	2.100	−0.026	0.980	−4.421	4.314
BMI, kg/m^2^	0.703	6.039	0.116	0.908	−11.856	13.262
Gender (1 = male, 0 = female)	−9.135	35.152	−0.260	0.797	−82.237	63.968
Coexisting hypertension (1 = yes, 0 = no)	−3.283	44.800	−0.073	0.942	−96.450	89.884
Anticoagulant type (1 = LMWH, 0 = indobufen)	42.181	41.749	1.010	0.324	−44.641	129.004
Disease type (reference: LS)						
LSS	34.305	40.133	0.855	0.402	−49.157	117.767
LDH	13.515	58.765	0.230	0.820	−108.695	135.724
Surgical segment (reference: three)						
One	−88.250	61.376	−1.438	0.165	−215.888	39.387
Two	−15.434	58.058	−0.266	0.793	−136.173	105.306

In the control group, results of the multivariate linear regression analysis—with IBL and PDV as dependent variables—demonstrated that the surgical segment was an independent influencing factor of IBL, while disease type was an independent predictor of PDV. None of the other independent variables emerged as independent predictors of either IBL or PDV. The results are presented in Tables 7 and 8.

**Table 7:** Multivariate linear regression analysis with IBL as the dependent variable in the control group

Variables	*B*	SE	*t*	*p*	95% CI	
					Lower bound	Upper bound
Constant	472.363	676.243	0.699	0.493	−943.030	1887.756
Age, year	5.809	5.277	1.101	0.285	−5.236	16.854
BMI, kg/m^2^	2.179	17.049	0.128	0.900	−33.505	37.863
Gender (1 = male, 0 = female)	−14.402	115.894	−0.124	0.902	−256.972	228.168
Coexisting hypertension (1 = yes, 0 = no)	66.525	114.110	0.583	0.567	−172.310	305.361
Anticoagulant type (1 = LMWH, 0 = indobufen)	15.386	105.871	0.145	0.886	−206.205	236.978
Disease type (reference: LS)						
LSS	27.700	115.373	0.240	0.813	−213.779	269.178
LDH	−154.356	140.473	−1.099	0.286	−448.368	139.657
Surgical segment (reference: three)						
One	−656.538	161.550	−4.064	0.001	−994.666	−318.410
Two	−286.382	169.301	−1.692	0.107	−640.735	67.970

**Table 8:** Multivariate linear regression analysis with PDV as the dependent variable in the control group.

Variables	*B*	SE	*t*	*p*	95% CI	
					Lower bound	Upper bound
Constant	180.791	230.564	0.784	0.443	−301.785	663.366
Age, year	3.164	1.799	1.759	0.095	−0.602	6.930
BMI, kg/m^2^	−3.533	5.813	−0.608	0.551	−15.700	8.633
Gender (1 = male, 0 = female)	−33.370	39.514	−0.845	0.409	−116.074	49.334
Coexisting hypertension (1 = yes, 0 = no)	6.505	38.906	0.167	0.869	−74.926	87.935
Anticoagulant type (1 = LMWH, 0 = indobufen)	−4.094	36.097	−0.113	0.911	−79.645	71.457
Disease type (reference: LS)						
LSS	59.199	39.336	1.505	0.149	−23.133	141.530
LDH	116.481	47.894	2.432	0.025	16.238	216.724
Surgical segment (reference: three)						
One	19.087	55.080	0.347	0.733	−96.197	134.372
Two	117.936	57.723	2.043	0.055	−2.880	238.751

**Figure 1:**
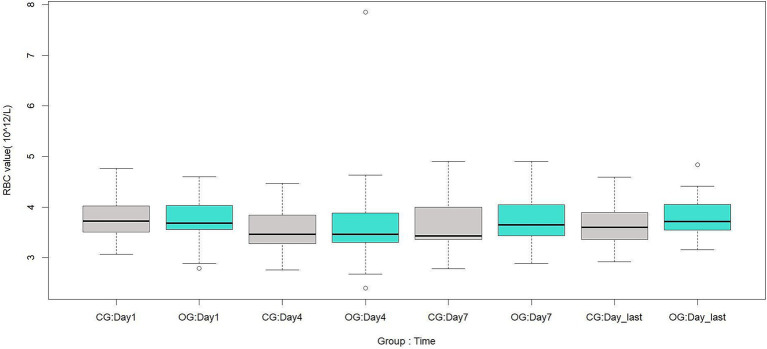
Comparison of postoperative RBC between the two groups, 10^12^/L. CG, Control group; OG, Observation group; Day1, 1st day; Day4, 4th day; Day7, 7th day; Day_last, Last-test day.

**Figure 2:**
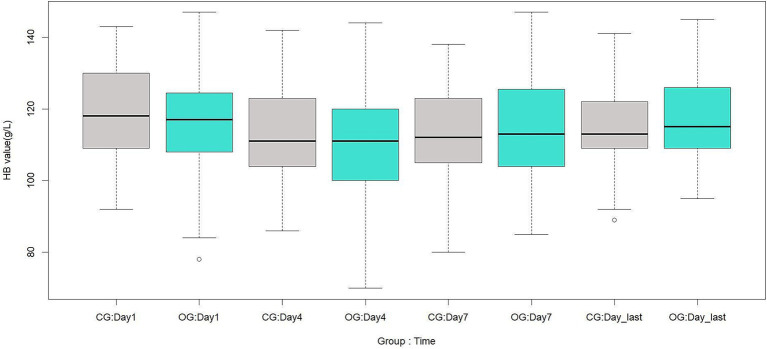
Comparison of postoperative HB between the two groups, g/L. CG, control group; OG, observation group; Day1, 1st day; Day4, 4th day; Day7, 7th day; Day_last, last-test day.

**Figure 3:**
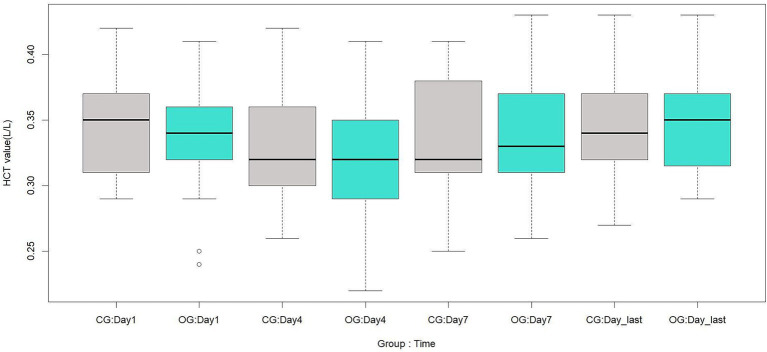
Comparison of postoperative HCT between the two groups, L/L. CG, control group; OG, observation group; Day1, 1st day; Day4, 4th day; Day7, 7th day; Day_last, last-test day.

There were no significant differences between the two groups in RBC, HB, or HCT on the 1st, 4th, 7th, and last-test days after surgery (*p* > 0.05). These results are shown in Table 9 and Figures 1–3.

**Table 9:** Comparison of postoperative HB, RBC and HCT between the two groups.

Groups	Observation group (*n* = 31)	Control group (*n* = 29)	*t*/*χ*^2^/*F*	*p*
HB, g/L				
Group			0.000	0.994
Time			0.132	0.941
Time*Group			0.454	0.714
1st day	115.387 ± 14.843	118.483 ± 13.540	−0.842	0.403
4th day	111.613 ± 17.435	112.759 ± 14.257	−0.278	0.782
7th day	115.839 ± 15.780	114.483 ± 13.574	0.356	0.723
Last-test day	117.323 ± 13.004	114.552 ± 12.802	0.831	0.409
RBC, 10^12^/L				
Group			0.826	0.364
Time			0.138	0.937
Time*Group			0.306	0.821
1st day	3.735 ± 0.445	3.766 ± 0.380	−0.286	0.776
4th day	3.46 [3.29; 3.97]	3.46 [3.28; 3.84]	−0.207	0.836
7th day	3.65 [3.42; 4.07]	3.43 [3.36; 4.00]	−0.710	0.478
Last-test day	3.71 [3.52; 4.07]	3.60 [3.36; 3.89]	−1.213	0.225
HCT, L/L				
Group			0.137	0.711
Time			0.223	0.880
Time*Group			0.342	0.795
1st day	0.338 ± 0.040	0.348 ± 0.039	−0.997	0.323
4th day	0.325 ± 0.049	0.329 ± 0.041	−0.356	0.723
7th day	0.33 [0.30; 0.37]	0.32 [0.31; 0.38]	−0.141	0.888
Last-test day	0.347 ± 0.039	0.342 ± 0.041	0.426	0.672

There were no significant differences between the two groups in postoperative PT, APTT, TT, FIB, PLT, or postoperative hospital stay (*p* > 0.05). These results are presented in Table 10.

**Table 10:** Comparison of secondary observation indicators between the two groups.

Groups	Observation group (*n* = 31)	Control group (*n* = 29)	*t*/*Z*	*p*
PT, s	11.803 ± 0.636	11.631 ± 0.873	0.878	0.384
APTT, s	28.581 ± 2.116	28.321 ± 2.173	0.469	0.641
TT, s	14.50 [13.90; 15.10]	14.72 [14.10; 15.40]	−0.873	0.383
FIB, g/L	3.048 ± 0.529	2.934 ± 0.402	0.937	0.352
PLT, 10^9^/L	205.032 ± 50.037	195.448 ± 50.521	0.738	0.464
Postoperative hospital stay, day	15 [11; 16]	12 [10; 15]	−1.176	0.240

## Discussion

Safety is a prerequisite for any clinical intervention. TXA reduces surgical bleeding by inhibiting fibrinolysis and stabilizing blood clots (15). Therefore, some scholars believe that TXA may lead to systemic inhibition of fibrinolysis and reduced thrombolytic activity, thereby increasing the risk of DVT (16, 17). Meanwhile, patients undergoing PLIF experience postoperative wound pain and require bed rest, which limits mobility and leads to poor venous return in the lower limbs. Moreover, the postoperative stress response and iatrogenic trauma induce a hypercoagulable state, while cardiovascular function and calf muscle pump function decline in elderly patients, all of which increase the risk of postoperative lower limb DVT (18). Therefore, DVT after PLIF is also a common clinical concern. Accordingly, the present study prioritized the assessment of safety. All surgeries were successfully completed, with good postoperative wound healing and no occurrence of DVT in either group. These findings indicate that TXA is safe and feasible for patients on preoperative anticoagulant therapy undergoing PLIF.

Surgery and blood loss can induce fibrinolysis and increased fibrinolytic activity in patients, which in turn leads to further blood loss. Intravenous administration of TXA can terminate this vicious cycle (19). Therefore, in this study, IBL and PDV were significantly lower in the observation group than in the control group. In general, the body maintains coagulation and anticoagulation systems, as well as fibrinolysis and anti-fibrinolysis systems, all of which are in a relatively balanced state (20). In theory, when anticoagulant drugs are administered before surgery, the anticoagulant system may be potentiated. When blood vessels are injured during surgery, the coagulation system is activated and blood clots are formed. Fibrin in blood clots can bind to plasminogen and activators, generating large amounts of plasmin, ultimately leading to clot dissolution and further bleeding (21). TXA reversibly binds to plasminogen, preventing its activation into plasmin, thereby exerting a hemostatic effect (8). However, in this study, there were no significant differences between the two groups in postoperative PT, APTT, TT, FIB, or PLT. This indicates that preoperative administration of TXA does not affect the coagulation system. To further identify the independent influencing factors of IBL and PDV, a multiple linear regression analysis was performed. The analysis revealed that the surgical level and TXA were independent influencing factors of blood loss, while age, BMI, gender, coexisting hypertension, disease type, and anticoagulant type were not. The influence of surgical segment on blood loss is clinically evident and well recognized. As the number of surgical segments increases, the complexity and trauma of the procedure also increase, which inevitably leads to greater blood loss. However, with advances in surgical techniques and the implementation of appropriate perioperative blood management strategies, an increasing number of patients undergoing multi-level PLIF have benefited from this procedure. Therefore, multi-segment PLIF is not considered a contraindication for surgery.

Different anticoagulant agents may exert distinct effects on blood loss, which merits consideration. Although a previous study reported no significant differences in the effects of LMWH and indobufen on IBL and PDV in PLIF (14), the present study further validated this finding by performing multivariate linear regression analysis in subgroups. The results revealed that anticoagulant type was not an independent influencing factor for IBL or PDV, which is consistent with the findings of that earlier study.

In general, a reduction in IBL can improve visualization of the surgical field and reduce the need for hemostatic maneuvers, which may ultimately shorten operative time. Moreover, with decreases in IBL and PDV, the number of blood transfusions would also be expected to decrease. However, in this study, although surgical time and the number of blood transfusions were lower in the observation group than in the control group, the differences were not statistically significant. There are three possible reasons for this finding. First, the body has considerable physiological reserves and homeostatic mechanisms. Although the control group had more bleeding, the blood loss remained within a compensatory range. Therefore, the blood loss was insufficient to necessitate a significant increase in blood transfusions. Second, as PLIF techniques have become more refined, surgical teams work more efficiently. Consequently, any additional time required for intraoperative hemostasis may not be sufficient to significantly extend the total operative time. Third, as a retrospective case–control study, this investigation has a relatively small sample size and may be subject to certain biases—an unavoidable limitation of such a design.

Theoretically, when surgical blood loss is reduced but blood transfusion requirements remain unchanged, postoperative anemia-related parameters should remain at relatively higher levels. However, in this study, there were no significant differences between the two groups in RBC, HB, or HCT at any postoperative time point. This is consistent with a previous finding that TXA did not increase RBC, HB, or HCT in patients undergoing two-level PLIF without anticoagulant therapy (22). There are three possible explanations for this finding. First, TXA is a hemostatic agent with a relatively short half-life. One study reported that the half-life of TXA is several hours, and the effective antifibrinolytic concentration in plasma is 1 μg / mL, which can be maintained for up to 16 h (23). As its plasma concentration decreases, its hemostatic effect diminishes accordingly. Second, preoperative anticoagulant therapy may enhance coagulation system activity during surgery, which could, to some extent, influence the aforementioned parameters. However, this remains a hypothesis, and the underlying mechanism requires further investigation. Third, the standardization of the PLIF treatment protocol ensured that both groups received the same perioperative medications and postoperative rehabilitation regimens. This may also explain the comparable postoperative hospital stay between the two groups.

### Limitations

Although this study yielded several meaningful findings, it has the following limitations. First, as a single-center, small-sample retrospective case–control study, this design is inherently susceptible to bias. This may have introduced selection bias and information bias, thereby limiting the generalizability of the findings. Second, while multivariate linear regression analysis within subgroups indicated that anticoagulant type was not an independent influencing factor for IBL or PDV, this finding warrants validation in studies with larger sample sizes. Additionally, the small sample size of the subgroups precluded further exploration of potential interactions between different anticoagulants and TXA. The interaction profile of these agents when used concomitantly remains to be elucidated in large-sample investigations. Third, this study also yielded certain negative findings, and the interpretation of these results should be approached with caution. These interpretations are largely speculative, as they lack support from mechanistic insights or objective indicator data.

## Conclusion

For patients undergoing PLIF who receive anticoagulant therapy within 1 week before surgery, a single preoperative 1 g dose of TXA is safe and effectively reduces IBL and PDV. However, given the limitations of this study, large-sample, multi-center, prospective randomized controlled trials incorporating more objective pharmacodynamic indicators are warranted to further validate these findings.

## Data Availability

The datasets presented in this study can be found in online repositories. The names of the repository/repositories and accession number(s) can be found in the article/supplementary material.
